# Proximity measurements for objective assessments of crew cohesion in an antarctic space analog environment

**DOI:** 10.3389/fpsyg.2026.1783846

**Published:** 2026-06-08

**Authors:** Mathias Basner, Ka’alana Rennie, Tyler M. Moore, Ruben C. Gur, Michael G. Smith, Jad Nasrini, Emanuel Hermosillo, Adrian J. Ecker, Victoria Schneller, Christopher W. Jones, Floris P. van den Berg, Bernd Johannes

**Affiliations:** 1 Unit for Experimental Psychiatry, Division of Sleep and Chronobiology, Department of Psychiatry, Perelman School of Medicine, University of Pennsylvania, Philadelphia, PA, United States; 2 Brain Behavior Laboratory, Department of Psychiatry, Perelman School of Medicine, University of Pennsylvania, Philadelphia, PA, United States; 3 European Space Agency,, Paris, France; 4 German Aerospace Center (DLR), Institute of Aerospace Medicine, Cologne, Germany

**Keywords:** astronauts, cohesion, confinement, isolation, proximity, teamwork

## Abstract

Astronauts will spend prolonged periods in an isolated, confined, and extreme (ICE) spaceflight environment on long-duration space exploration missions. Continuous confinement in close quarters with a few crewmembers will require high levels of crew cohesion to promote mission success and crewmember wellbeing. In the past, the most common measure of crew cohesion were surveys, which tend to have high participant bias. More recently, researchers have analyzed video recordings that provide detailed proximity information but are time consuming to score and prone to interobserver bias. Here, we measured crewmember proximity as a surrogate measure of crew cohesion with proximity sensors worn by crewmembers during two Antarctic 10–14 months winter-over missions (*N* = 13 in 2015 and *N* = 12 in 2016). These sensors recorded instances of crewmembers being close in distance to each other, allowing for the identification of trends in crewmember interactions and crewmember withdrawal. We demonstrate that proximity is an unobtrusive, objective, and easy to obtain surrogate measure for crew cohesion that reveals systematic changes with time in mission. This includes a decrease in crew cohesion resulting from more time spent alone as each mission progressed, and higher average crew cohesion scores in Year 1 than in Year 2. Findings from this study could be used to monitor sudden or gradual withdrawal from other crewmembers and provide insights unobtrusively on crew cohesion without compromising crew autonomy.

## Introduction

Exploration-class space missions will require humans to live in isolated, confined, and life-threatening environments for prolonged periods of time (Patel et al., 2020). Using conventional propulsion and accounting for celestial mechanics, the standard reference mission to and from Mars is estimated to last at least 500 days, well beyond the duration astronauts and cosmonauts have remained confined in a spacecraft. These missions will also require greater crew autonomy than currently experienced in spaceflight (Kanas et al., 2009). NASA’s evidence-based review of the behavioral health risks to crew and mission success during exploration spaceflight concluded they were among the most serious unmitigated risks to such missions (Slack et al., 2009), a view shared by the Aerospace Medical Association (Baisden et al., 2008).

Antarctic research stations are considered a high-fidelity analog for long-duration space missions due to their, (1) complex logistical operations, (2) harsh threatening environmental conditions such as extreme cold, altered photoperiod, low humidity, isolation, and confinement, and (3) multicultural populations of researchers with educational backgrounds comparable to those of astronauts. Similar to astronauts, winter-over participants in Antarctic stations have anecdotally reported motivational decline, somatic complaints like sleep disturbance, headaches, as well as decrements in mood and morale (Harrison and Connors, 1984), among other symptoms. These symptoms have been referred to as Winter-over Syndrome (Palinkas and Suedfeld, 2008). Furthermore, Antarctic environments, like spaceflight, require crewmembers to spend prolonged periods of time in confined space with the same group of people, which increases the likelihood of interpersonal conflicts and its associated negative effects on crew cohesion, potentially compromising mission success (Marcinkowski et al., 2021).

Humans are social animals. Contact and communication with other human beings are important for social development and general wellbeing, but on the other hand, opportunities to avoid contact can be equally important to prevent the escalation of conflicts within social groups, making both social contact and its avoidance are likely important for crew cohesion (Bell et al., 2025). Exploration-class missions will require data on a small group of crew members who spend prolonged periods of time in isolated, confined, and stressful environments, while working even more autonomously than is usual in current 6–12-month missions aboard the International Space Station (ISS).

Some studies have investigated crew cohesion in ground-based spaceflight simulations (e.g., SFINCSS, Mars500, ISEMSI/EXAMSI) and in space (Sandal et al., 1995; Kanas et al., 1996, 2007; Johannes et al., 2015). Questionnaires are the standard method for assessing group structure but are subject to several kinds of bias (e.g., cultural bias, recall bias). Methods to objectively, continuously, and unobtrusively investigate group cohesion are therefore warranted (Johannes et al., 2015). Although a step in this direction, the analysis of observations or video recordings (Tafforin, 2013) is still time consuming and prone to inter-observer variability in the quantification of crew interactions.

To objectively assess crew cohesion during extended stays in ICE space analog environment, we continuously and unobtrusively measured crew cohesion in *N* = 25 subjects during two 1-year Antarctic winter-over missions in the French/Italian Concordia station. We used low-energy Bluetooth^®^ Smart devices that recorded proximity to each other, which allowed us to investigate how much time each crewmember spent in close proximity with other crewmembers across the winter-over. This was a proof-of-concept study, aiming to measure proximity in an Antarctic research station to investigate how much time individual crewmembers spent with other crewmembers. We hypothesized that there would be large inter-individual differences in how much time individuals spent with the rest of the crew, and that this time would change as the mission progressed.

## Materials and methods

We investigated neurostructural, cognitive, sleep, activity, crew cohesion, and habitat use changes in researchers and staff overwintering in Concordia station, which is operated by the French Polar Institute (Institut Paul Emile Victor, IPEV) and the Italian Antarctic Research Programme (PNRA).

This manuscript focuses on changes in crew cohesion (changes in brain structure, sleep, and cognitive performance were presented elsewhere (Roalf et al., 2025). The European Space Agency (ESA) entered into a cooperation with IPEV and PNRA in 2001 to use Concordia station for preparatory activities related to future human space exploration missions. ESA deploys a research medical doctor (MD) for Concordia missions, who is responsible for the conduct of the scientific projects. Each Concordia winter-over crew typically consists of 12–14 subjects. We investigated a total of 25 crew in two consecutive 10–14 months winter-overs: 2015 (year 1) and 2016 (year 2; see 2.2 below for details). During the winter-over, we continuously recorded wrist-actigraphy and proximity information with a single device to monitor changes in sleep-wake behavior, crew cohesion, and habitat use with time-in-mission. This was a proof-of-concept study for using this proximity technology as a proxy for crew cohesion across two Antarctic winter-overs.

### Concordia station characteristics and operation

Concordia station is located at Dome C on the Antarctic Plateau at 75° 06’ S, 123° 23’ E and has a size of 1,500 m^2^ (see Supplementary Figure 1). This location is 685 miles inland from Dumont d’Urville (French coastal station) and 750 miles inland from Mario Zucchelli Station at Terra Nova Bay (Italian coastal station). This area is considered one of the most hostile places on Earth. Access to Concordia station is limited to the austral summer (November to February) due to the extreme weather conditions (average winter temperature −76 °F/−60 °C). Concordia station can host up to 16 crewmembers for a winter-over. The winter-over teams are selected through medical/psychological screening by IPEV and PNRA. The over-winter crew stays for at least 10 months and up to 14 months in Concordia station.

The main task of crewmembers is not to serve as test subjects, and participation in research projects is voluntary. Also, ESA does not allow any monetary compensation for participation in research studies. Therefore, experiments must be non-invasive and consume little time to assure high participation and adherence rates. As ESA implements an average of eight experimental protocols during any given winter-over period, and a maximum of 2 h per week (per subject) are available for research purposes, the time available per project and subject is limited to 15 min per week.

### Research participants

A total of 25 participants were recruited for the study. Of these, 13 (3 female) and 12 (2 female) participants over-wintered at Concordia station in Year 1 and Year 2, respectively. The crew’s average age (mean ± SD) was 34.2 ± 11.1 years (range 24–56 years) in Year 1 and 39.9 ± 12.2 years (range 22–62 years) in Year 2, and thus comparable to astronaut demographics (Smith et al., 2020). Concordia crews are mainly composed of researchers, but also include a cook, engineers for maintaining the station, and a medical doctor. A second medical doctor was hired by ESA and was responsible for research project implementation during the winter-over.

The study was approved by the Institutional Review Board at NASA’s Johnson Space Center (protocol #1323) and by Ärztekammer Nordrhein, Cologne, Germany. All subjects provided written informed consent prior to their participation in the study.

### Proximity measurement

The Concordia crew were asked to continuously wear an actigraph (Actigraph Link, Actigraph, Pensacola, FL) on the wrist of the non-dominant arm. A small minority of crewmembers decided to wear the watch at a different location (e.g., ankle, waist belt), so that it did not interfere with manual labor. The Actigraph Link monitor is the size of a wristwatch and weighs only 14 g. It measures accelerations associated with wrist movements in three orthogonal axes and stores information at a rate of 30 Hz. Actigraphy is a non-invasive method to gather objective sleep-wake data over prolonged periods of time. The methodology has been validated to provide reliable estimates on wake and sleep times and activity levels (Ancoli-Israel et al., 2003; Basner et al., 2013).

A unique feature of the Actigraph Link is that it also registers other devices in its proximity. Thus, a single device was used to investigate both crew sleep-wake behavior and crew proximity, decreasing study participant burden. The devices use low-energy Bluetooth^®^ technology to detect other devices in their vicinity. They automatically log other detected devices at a once per minute rate with the received signal strength indicator (RSSI) in decibels as a measure of proximity. The detection range varies greatly - from 10 to 20 m indoors up to 100 m outdoors (according to the manufacturer) - depending on the nature of the environment (e.g., number and material of walls), but it is expected to be consistent between devices. In our own testing, signal strength was found to be an unreliable surrogate of proximity, and we thus decided against using it for data analysis. Instead, we treated proximity as a binary variable (i.e., encounter yes/no). This is further explained below.

In Year 1, the watches could only be programmed as either beacons or receivers. ESA’s research MD thus switched the watches from receiver to beacon and vice versa on a weekly basis according to a pre-defined schedule that assured that each crewmember was able to “see” each other crewmember approximately the same amount of time across the whole winter-over. In Year 2, watches could be programmed to simultaneously send and receive proximity data. However, due to a hardware/software bug that was detected during data analysis, watches programmed as both beacons and receivers would unforeseeably stop sending and receiving proximity data. Because of this bug, we lost 2.6% of the proximity data in Year 2. With the proximity feature turned on, the rechargeable actigraph batteries lasted for 10 days. This was not a problem in Year 1, as actigraphs were exchanged for fully charged watches on a weekly basis. In Year 2, the crewmembers charged their watches during lunchtime in the cafeteria on an as-needed basis (i.e., when the battery life indicator on the watch indicated a low charge). At the time of data collection, there was, to our knowledge, no publications validating this feature. Since then, Dlugonski et al. (2019) used a device from the manufacturer with the same feature to validate proximity detection in several conditions related to exercising together. They concluded that, while there were limitations, these devices were a reliable method for detecting shared physical activity.

### Statistical analysis of proximity data

In Year 1, two crewmembers had a<50% actigraph wear time during daytime (defined as 9 a.m. until 9 p.m.) and were excluded from data analysis. The remaining crewmembers had on average 88.0% wear time during daytime (range 59.1%–98.8%). In Year 2, two crewmembers stopped wearing the actigraph 4 months into the mission and were excluded from data analysis. The remaining crewmembers had on average 83.3% wear time during daytime (range 75.6%–93.7%). Thus, 11 crewmembers from Year 1 to 10 crewmembers from Year 2 contributed to data analysis, yielding a total analytic sample of *N* = 21 crewmembers.

For crew proximity analyses, we first determined whether two crewmembers were “at risk of seeing each other,” and if so, whether or not the watches actually detected each other. For two crewmembers to be at risk of seeing each other, both had to be awake and, for Year 1 only, one watch had to be programmed as a beacon while the other had to be programmed as a receiver, or vice versa. We investigated all possible dyads of crewmembers (55 dyads in 2015 and 45 dyads in 2016). For each dyad, we calculated the percentage of time at least one of the two actiwatches indicated proximity to the other actiwatch relative to the time they were at risk for seeing each other (see above). These calculations were performed for the whole winter-over as well as separately for each week of the winter-over (the latter for Year 2 only). We also averaged the percentage of time a crewmember spent with other crewmembers across all crewmembers and the winter-over. Furthermore, the proportion of time a crewmember spent (a) alone; (b) with one other crewmember; and (c) with two or more other crewmembers was calculated. We also derived a measure of cohesion - defined as the proportion of actual contacts relative to all possible contacts - for each crewmember across the 10 months of the winter-over. This value ranges from zero (crewmember spent all time alone) to one (crewmember spent all time with all other crewmembers) and is again based on time and risk of seeing other crewmembers. Values of individual crewmembers were averaged to derive a time course of crew cohesion that individual crewmembers could be compared to. Additionally, the structure of relationships among crewmembers was modeled using factor analysis (least-squares extraction) with oblique (oblimin) and bifactor (Schmid-Leiman) rotations (Schmid and Leiman, 1957; Reise et al., 2010). The input was a square symmetric matrix with rows and columns representing participants, and values indicating interaction likelihoods. For example, cell [1,2] indicated the proportion of time participants 1 and 2 spent together out of all time they were “at risk” of seeing each other. The diagonal of the matrix was therefore 1.0 (100%) for all, indicating amount of time spend with oneself. The factor analysis of this matrix produced loadings associated with each person, where the factors on which they loaded could fairly be considered “cliques” of people. If two participants loaded highly on factor 1, for example, it indicated they were part of the same “clique.” For the bifactor model, one of the factors was a “general” factor, where a strong loading on that factor indicated that s/he was generally sociable regardless of clique. Several indices have been developed (Rodriguez et al., 2016) for quantifying the strength of the general factor in a bifactor model, and here we used omega-hierarchical (ω_h_),which is calculated as the squared sum of loadings on the general factor divided by the sum of the sums of squared loadings on the specific factors (plus the uniquenesses)—see Eq. 6 in Rodriguez et al. (2016). It is interpreted as the strength of the general factor relative to all factors combined, which, in this case, can be interpreted as the cohesion of the crew independent of cohesion within its cliques.

After the winter-over, ESA’s research MD was asked to fill out the “Individual-referent” section of the group living skills (GLS) survey (Landon et al., 2024). For each crewmember (including himself/herself), each research MD had to rate agreement with the following five statements on an 11-point Likert-scale with the anchors “strongly disagree” (0) and “strongly agree” (10): “uses humor appropriately”; “appreciates others’ knowledge, skills, and abilities”; “clean and tidy with personal items”; “clean and tidy with work items”; “if I were doing another mission, I would want her/him as a crew mate.” The group living score was then correlated with average time spent with other crewmembers and with scores (loadings and ω_h_) derived from the factor analyses.

## Results

**FIGURE 1:**
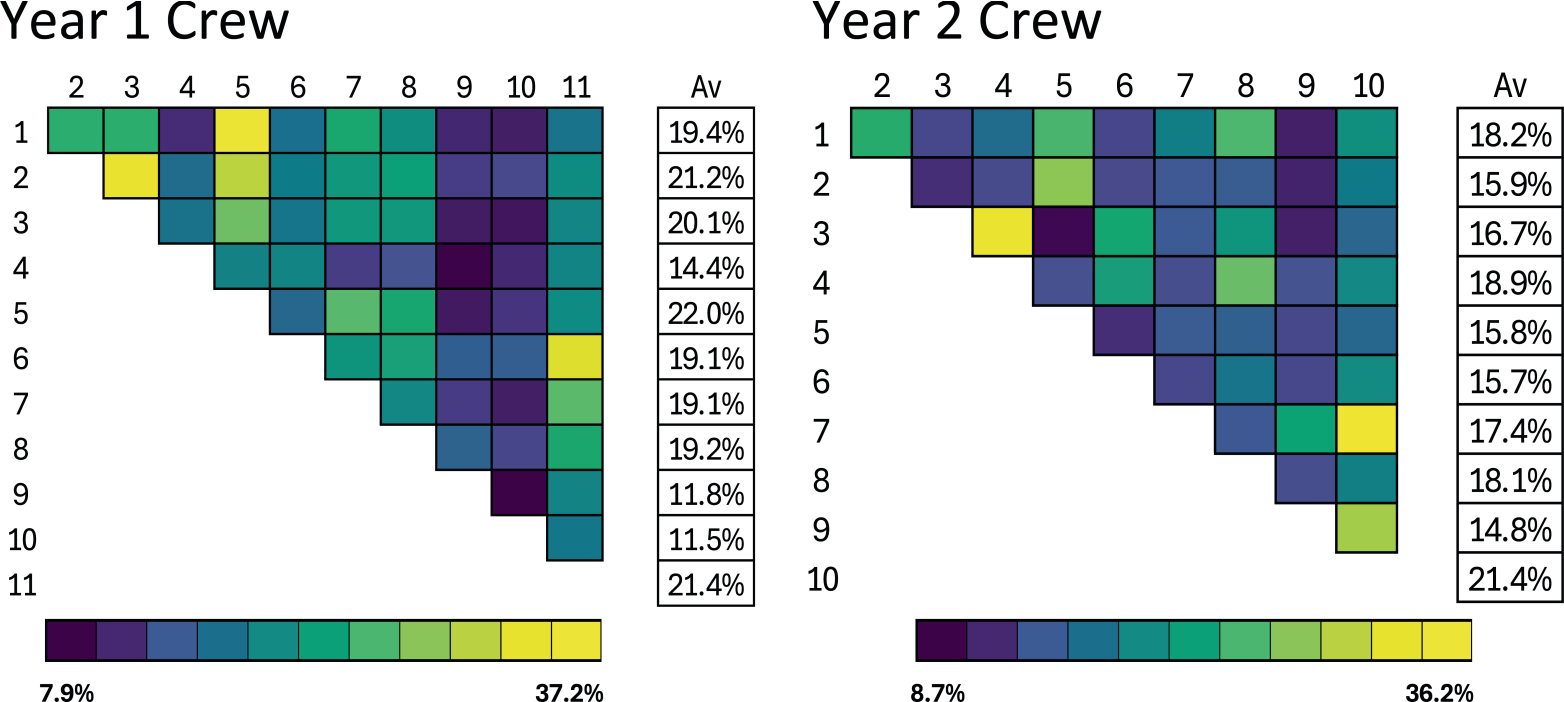
Percentage of time two crewmembers spent in proximity to each other relative to the total time at risk of detecting each other. The numbers next to and above the heat map reflect crewmember IDs. The color gradient below each heatmap represents the spectrum of percent of time spent in proximity ranged from the least amount of time (purple) to the greatest percentage of time (yellow). Av, average value across all crewmembers.

Figure 1 illustrates the percentage of time two crewmembers spent in proximity to each other relative to the total time at risk of detecting each other over the whole winter-over. For example, in 2015 crew #1 and #5 spent the greatest proportion of time (37.2%) in proximity to each other, while crew #4 and #9 spent the smallest proportion of time (7.9%) in proximity to each other. On average, crewmember #10 spent the least amount of time in proximity of all other crewmembers. In 2016, crew #3 and #4 spent the greatest proportion of time (36.2%) in proximity to each other, while crew #3 and #5 spent the smallest proportion of time (8.7%) in proximity to each other. On average, crewmember #9 spent the least amount of time in proximity to all other crewmembers.

**FIGURE 2:**
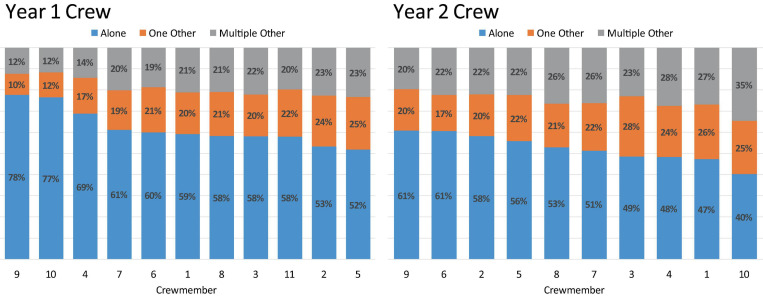
Allocation of time crewmembers spent alone and with other crewmembers. Percent of time spent alone, in proximity of one other crewmember, or in proximity to two or more other crewmembers relative to the time at risk of seeing other crewmembers.

The percentage of time that individual crewmembers spent alone or with one or multiple other crewmembers is shown in Figure 2. In Year 1, crewmembers #9 and #10 spent more than 75% of their time at risk alone, while crewmembers #2 and #5 spent just over 50% of their time at risk alone. In Year 2, crewmember #9 spent the highest proportion of their time at risk alone with 61%, while crewmember #10 spent only 40% of their time at risk alone. It should be noted that the statistics presented in Figure 2 cannot be directly compared between years since actigraphs could only be programed as beacons or receivers, but not both, in Year 1 (i.e., the opportunity to detect other actigraphs was lower in Year 1 than in Year 2). This is reflected in the average percent time spent alone across crewmembers, which was higher in Year 1 (62.1%) compared to Year 2 (52.4%).

**FIGURE 3:**
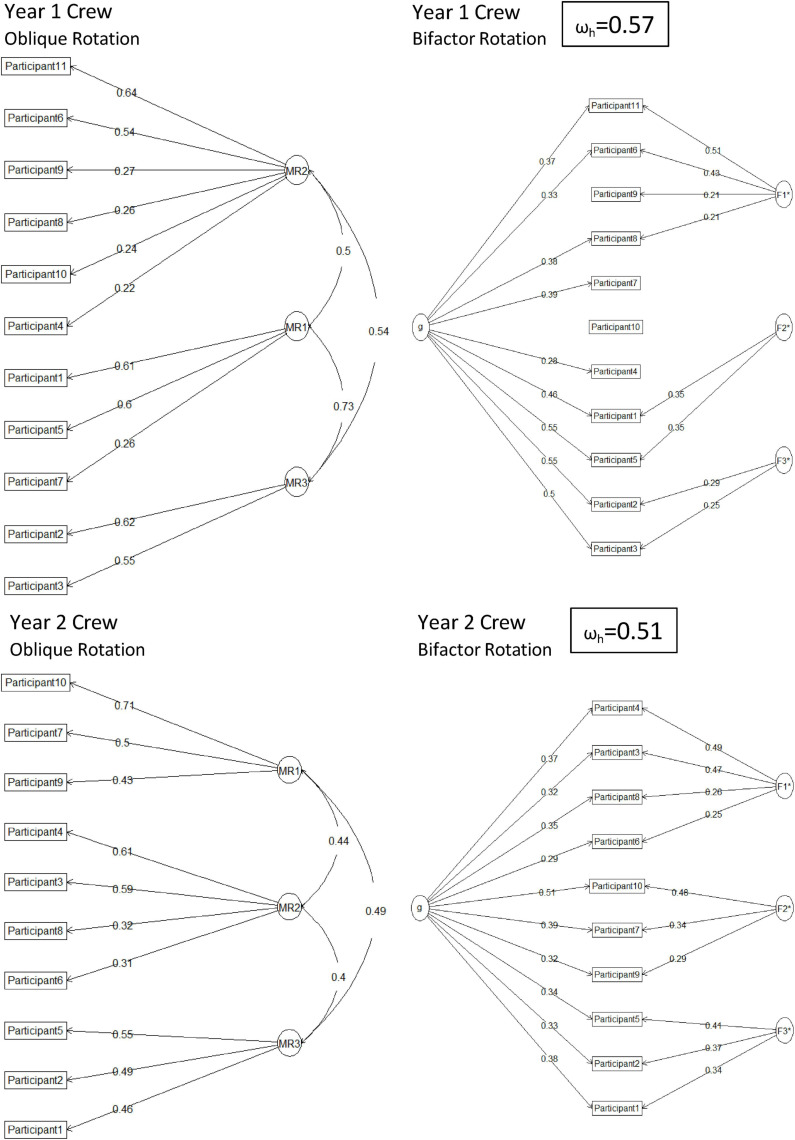
Factor analysis of crew proximity data. Numeric values reflect factor analysis loadings associated with each person. Boxes labeled participant reflect individual crewmembers. MR, short for “Minimum-residual, Rotated,” indicating the extraction method (“minimum-residual”; a.k.a., “least-squares”) and the fact that the solution is rotated.

Figure 3 shows the interaction structures for oblique and bifactor rotations for both study years. In the oblique solutions (left of Figure 3), the strengths of the associations between participants and their respective factors can be interpreted as correlations, where squaring them will give the proportion of variance in the individual’s behavior that is predictable based on group membership. The inter-factor correlations (designated “MR”) indicate the strength of the connections between groups. Taken together, ω_h_ is a rough indicator of overall group cohesion, which was higher in 2015 (ω_h_ = 0.57) compared to 2016 (ω_h_ = 0.51).

For bifactor interaction structures shown on the right of Figure 3, as in the oblique solution described above, associations between participants and their respective factors can be interpreted as correlations. However, in contrast to the oblique solution, the bifactor model posits a general factor (“g”) linking all participants directly to “overall cohesion.” This is an advantage of the bifactor model, because it allows one to compare direct links to overall cohesion, where it could only be inferred in the oblique model. Further, because these direct links are modeled, one can use an established measure of g-factor strength (McDonald’s ω_h_). In the present case, ω_h_ was 0.57 for the Year 1 crew and 0.51 for the Year 2 crew. There are no well-established benchmarks for what constitutes a high or low ω_h_, but it is worth noting that the values > 50% (>0.50) indicate that the majority of covariance (among people, in this case) is attributable to overall cohesion rather than clique-based cohesion.

In Year 1 and consistent with Figures 1, 2, crewmembers #9 and #10 emerged as contributing very little to overall crew cohesion, while crewmembers #2 and #5 most strongly contributed to overall crew cohesion. In Year 2 and again consistent with Figures 1, 2, crewmembers #6 and #9 stood out as contributing the least to overall crew cohesion, while crewmember #10 most strongly contributed to overall crew cohesion. That crew cohesion was lower in Year 2 relative to Year 1 was consistent with subjective reports by the research MDs after the respective winter-overs, although data between winter-over years should be compared with caution due to the different methodological approach in proximity measurements.

**FIGURE 4:**
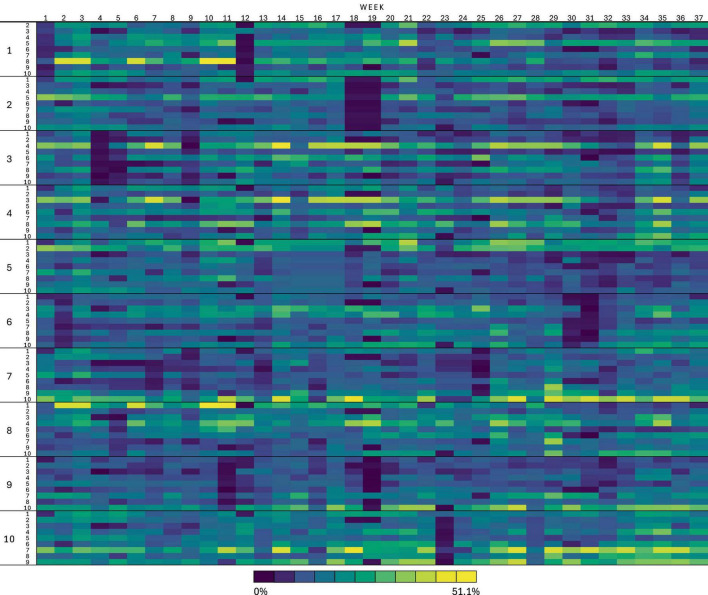
Heatmap of crewmember proximity across the Antarctic winter-over (Year 2 crew). The heatmap displays the percent of time two crewmembers spent in proximity to each other relative to the total time at risk of detecting each other across the 37 weeks of the mission for the Year 2 crew. C1 and C2 represent two crewmembers of a dyad. Mission week 1 started on 16 February 2016. The color gradient below the heatmap represents the spectrum of percent of time spent in proximity ranged from the least (purple) to the greatest (yellow) amount of time. Dark purple rectangles reflect either no proximity detection or actigraph non-wear by one or both crewmembers.

Crew cohesion is a dynamic process that was expected to change over the course of the winter-over. This is illustrated for the Year 2 crew in Figure 4. For example, crewmembers #1 and #8 spent more time in close proximity up until mission week 12, with a decreasing trend in time spent in close proximity across the rest of the mission. Factor analysis results by mission half can be found in Supplementary Figures 2, 3. They suggest lower crew cohesion in the second compared to the first mission half in both Year 1 and Year 2.

**FIGURE 5:**
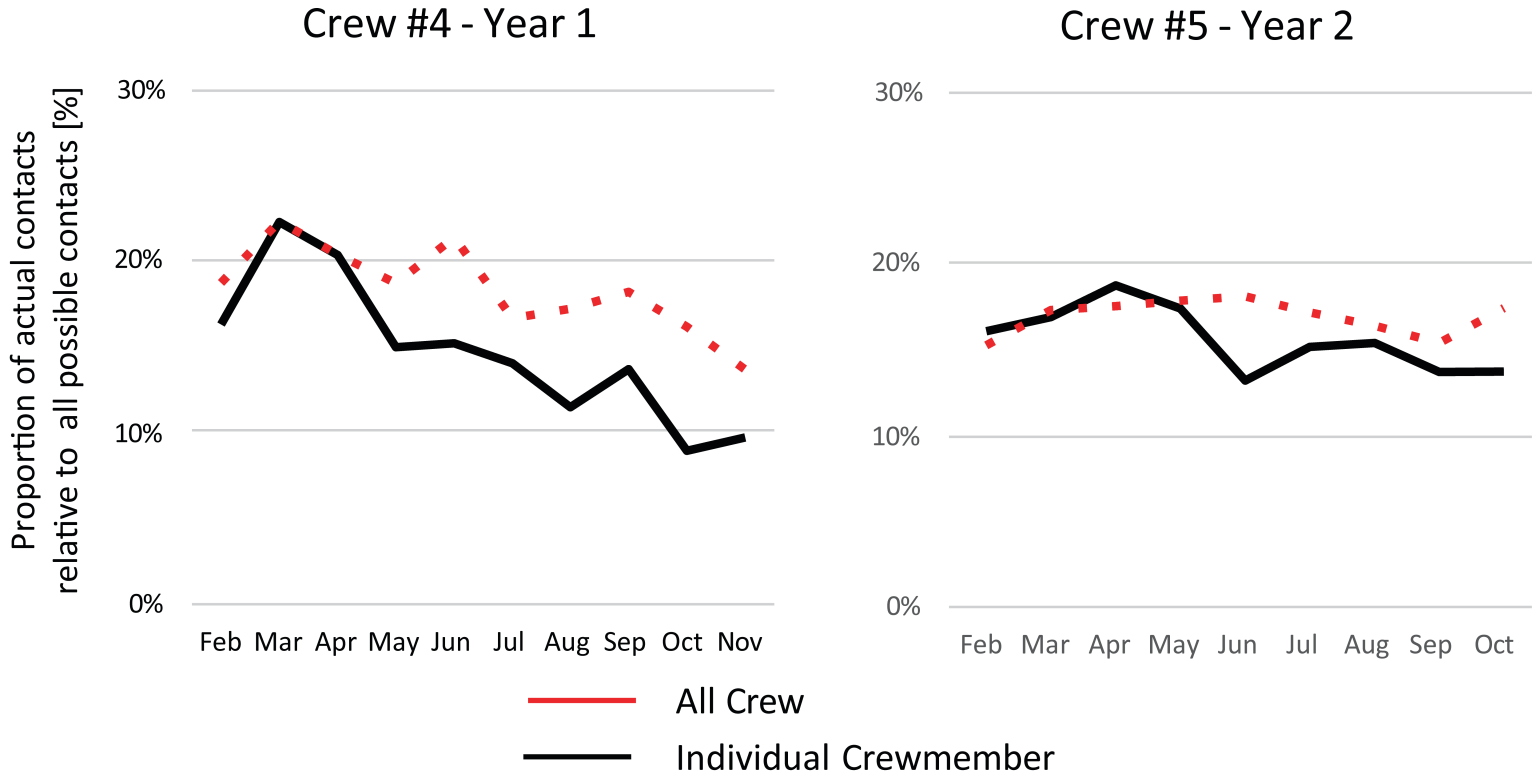
Representative plot comparing “togetherness” of two crewmembers against all crewmembers in Years 1 and 2. A measure of “togetherness” for one crewmember across the study period from each study year is shown in black against the average across all crewmembers of that year (red dotted line).

Figure 5 shows how average crew cohesion, defined as the proportion of the number of actual crew contacts relative to all possible contacts, changed across the mission for the Year 1 and the Year 2 crew (red dotted lines). This analysis again demonstrates that average crew cohesion was slightly higher in Year 1 compared to Year 2. Figure 5 also shows how the behavior of an individual crewmember is deviating from the behavior of an average crewmember. The figure on the left suggests that crewmember #4 (Year 1) was interacting with the crew at the average level for the first 3 months of the mission but then withdrew from and spent less time with the crew. The figure on the right suggests that crewmember #5 (Year 2) was interacting with the crew at the average level for the whole mission except for June. During mid-June, the crew has one week off and is celebrating mid-winter-over. Graphs for all crewmembers can be found in Supplementary Figures 4, 5.

**FIGURE 6:**
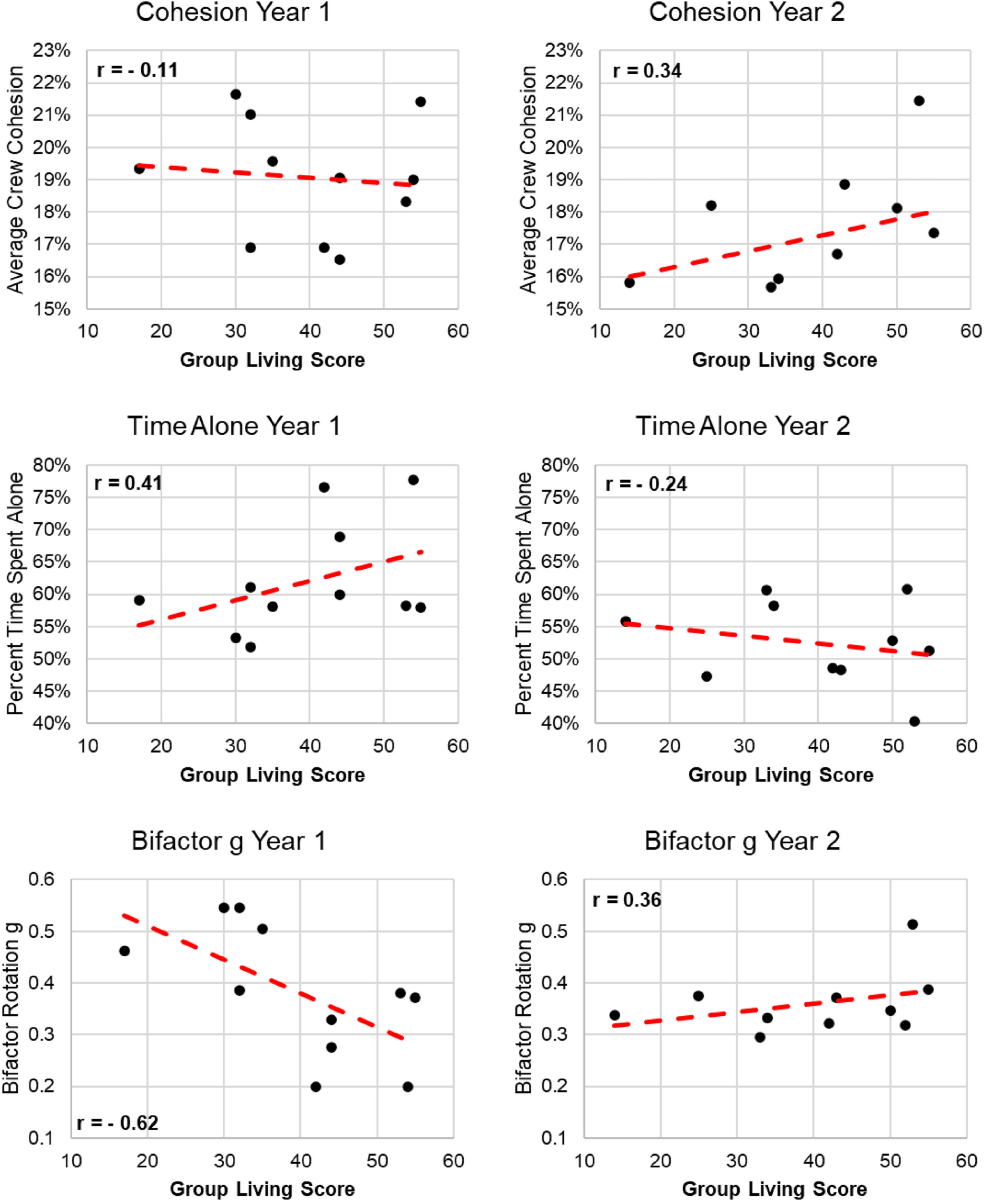
Pearson correlations between the group living score and three measures of crew cohesion for Year 1 and Year 2 separately.

Figure 6 shows Pearson’s correlations between the group living score (higher values = better) and three different measures of crew cohesion for both study years: (1) average crew cohesion (see Figure 2); (2) time spent alone (see Figure 3); and (3) personal loading on the g factor derived from factor analysis with bifactor rotation (see Figure 4). These correlations are inconsistent between years 1 and 2, i.e., if a correlation was positive in 1 year it was negative for the other year and vice vera. Also, correlations were low in general with the exception of the year 1 correlation for Group Living Scores and bifactor g scores. Here, R^2^ was 0.38, but the relationship was negative, i.e., higher g scores were associated with lower Group Living Scores. These scores were obtained from the Group Living Scale that was still under development when this study was performed but has since been fully validated (Landon et al., 2024).

## Discussion

Given the importance of social support and crew cohesion to the success of long-duration spaceflight missions where astronauts will spend prolonged periods in isolation and confinement, this study objectively measured crew proximity using wrist-worn actigraphy devices continuously across two winter-over campaigns at the Antarctic Concordia research station in *N* = 21 crewmembers. We found that crew proximity changed over time and that lower group crew cohesion was observed in the second compared to the first half of winter-over campaigns in both crews investigated in Years 1 and 2. While we can only speculate about potential reasons for this, decreases in crew cohesion seem to be a common feature in ICE environments as similar results were found by Johannes et al. (2015). The increase in time alone could potentially be a coping mechanism to living in an ICE environment like Antarctica for a prolonged time (Palinkas, 1992). Our findings suggest that wrist-worn actigraphs with a proximity feature can be used to assess crew cohesion in an unobtrusive and objective fashion. These devices provide continuous data with minimal participant burden, since crews are generally already wearing smartwatches. Also, there are fewer data privacy concerns compared to continuous video surveillance. Similar to this study, Johannes et al. (2015) used wireless sensors to track crew relations and physiological changes in a Mars 500 study. While they obtained significant results, finding crew cohesion declined over the whole mission time, subjects only wore the sensors twice a week, limiting the number of interactions they were able to observe and time resolution for predicting changes in crew cohesion.

In both years of this study, inter-individual differences in the amount of time spent alone and interacting with other crewmembers were evident. In Year 1, the crewmembers who had the strongest dyadic relationships also loaded most heavily on overall crew cohesion, while the two crewmembers who spent the least amount of time with others (Participant #9 and #10) contributed little to overall crew cohesion. While proximity measures do not inform about the nature and valence of an interaction, monitoring changes in an individual’s group participation could provide insight into crew cohesion. Especially decreases in crew proximity of individual crewmembers could be valuable early warning signs of interpersonal conflicts or emerging mental health issues that can be addressed early before their escalation. In this respect, while proximity was sometimes fixed by mealtime schedules or where and who someone worked with, these instances can be analyzed as an individual’s change in behavior. Much of our analysis was based on comparing individual measures of cohesion and proximity to other individual crewmembers as well as the whole group. For example, Figure 1 represents a matrix of time a subject spent with each other crewmember and overall. The cell representing a specific crewmember and their work partner can be interpreted differently from other cells. Objective proximity measurements can also be a potentially valuable research tool for investigating how crew interactions affect crew performance and mission success in space analog environments, and how crew cohesion is associated with cognitive performance and physiological changes throughout a mission. Even when team members have a shared goal and passion, conflicts, especially work related, may negatively impact team effectiveness (Kjærgaard et al., 2022).

Based on personal accounts observed by Palinkas (1992), crewmembers in Antarctic stations wished for alone time to avoid social tensions like a constant lack of privacy and gossip leading to most crewmates spending much of their leisure time alone. However, excessive isolation with an absence of social support was also detrimental (Palinkas, 1992). Previous research suggests that, the distinction between personal and task related time blurs during time in isolated and confined environments (Migaki et al., 2025). If true, it underscores the importance of crew cohesion for mission success since they may be interchangeable factors to the crew. The Self-reported measures of social support suggest that as time progresses in space analogs, individuals perceived social support is slightly higher among fellow crew members compared to perceived social support from family/peers (Bell et al., 2025). Since those embarking on exploration-class missions will have fewer opportunities for interaction with their usual support system, individuals may rely on fellow crewmembers in their absence, enforcing the importance of social support for wellbeing.

In addition to isolation, crewmembers in exploration-class missions will face life-threatening situations, which could increase stress and worsen mental health. With increased levels of stress and physical exhaustion, individuals may be more likely to instigate conflicts that disrupt crew cohesion (Basner et al., 2014). There are promising new well-being-supportive technologies for deep space exploration like artificial intelligence (AI) algorithms and virtual reality (VR), but unlike proximity measures, at their current stage of development they may be subject to biases surrounding neurodivergence and race, among others, which could further harm astronauts from underrepresented backgrounds in deep-space (Smith et al., 2023). An advantage of the actigraphy watches is that since crewmembers constantly wear them, data are continuously collected and allow for monitoring any concerning trends (i.e., crewmember social withdrawal from the group) before interpersonal issues or affective disturbances become too severe. Using proximity data, we were able to represent a value of “togetherness,” defined as the proportion of actual contacts relative to all possible contacts for all crewmembers. For some crewmembers (like crewmember #4 in Year 1), there is a clear deterioration in their proximity habits throughout the winter-over and compared to the rest of their crew. If crewmember 4 were on a mission to Mars, mission control could have noticed and addressed the deviation from the norm early on.

As discussed in the introduction, one of the main methods for assessing group structure in ICE environments has been questionnaires, but they are prone to several biases (Johannes et al., 2015). Analyzing video footage of individuals in space analogs is another common method for measuring crew cohesion but at the current stage time consuming (Tafforin, 2013), although automatic analysis of video feeds with computer vision and machine learning represents promising new avenues for this type of analysis. We used a wearable sensor to measure proximity which is unobtrusive and less biased for measuring crew cohesion compared to both questionnaires and video analysis. Still, future research could aim to utilize other upcoming technologies alongside proximity measures for a better understanding of crew cohesion. One of the more novel wearable sensors in team dynamic research is the “sociometric badge,” a wearable sensor that collects data on movement, audio, and social interactions (Zhang et al., 2018). While this is a promising tool that records a variety of data helpful for interpreting crew cohesion, we were unable to use these sensors in our own research due to technical difficulties. As previously discussed, proximity measures are useful for monitoring and early identification of concerning trends by looking at isolating behaviors. Contractor and DeChurch (2025) created a dashboard using computational modeling, which includes two models that predict social relationships and shared cognition. This is arguably a useful tool for monitoring relationships and early identification of deteriorating relationships. Future research could include such computational models to validate proximity as a surrogate for crew cohesion. Another area for future investigation is crewmember habitat utilization patterns, since proximity sensors were not only worn by crewmembers, additional sensors were placed around the station. In general, future research could focus on further evaluating associations between this proximity methodology and team cohesion and performance assessments. In this way, proximity measures could complement other methodologies, adding an objective component to self-report measures of cohesion.

### Limitations

While actigraphs provide an unobtrusive measure of both sleep-wake activity and proximity, these devices were not initially designed to measure the latter. To connect to other watches, they use low-energy Bluetooth technology which automatically logs other devices nearby, but the range drastically varies from 10 to 20 m indoors to 100 m outdoors (according to the manufacturer). Since the range in signal strength was unreliable during testing, we decided against using this as a measure of proximity and instead measured proximity as a binary outcome (i.e., watches came in contact or not, see p. 4). Additionally, we were unable to compare measurements between study years. In Year 1, actigraphs had to be reprogrammed as beacons or receivers on a weekly basis, as they could not simultaneously act as beacons and receivers. In Year 2, actigraphs could be programmed as both beacons and receivers, but due to a bug these watches sometimes unpredictably stopped receiving proximity data, although the resultant data loss was modest (2.6% of the proximity data that year). Due to these bugs, the company has since discontinued the proximity feature of their actiwatches. To further validate proximity as a measure of crew cohesion, we analyzed responses from the group living questionnaire, a survey designed to measure teamwork and likability of an individual based on scores given to them by colleague(s) they live with (Landon et al., 2024). Outside of the proximity technology, this scale was the only other source of data we used to develop an understanding of cohesion between crewmembers at the station. It primarily focuses on individual characteristics rather than overall team function which may explain discrepancies in scores between years.

Also, scores for the group living scale may not reflect collective crew opinions of an individual since only one crewmember, the research physician, completed these surveys retrospectively each year. The discrepancies in trends between Year 1 and 2 (see figure 6) may be a result of recall bias, or that group living on an individual level only captures one aspect of crew cohesion and proximity. Because of this, future studies should use diverse measures of crew cohesion in general, and have all crewmembers complete the group living scale more specifically. This would allow for a more thorough evaluation of proximity as a measure of crew cohesion.

Additionally, while all crewmembers participated in this study, two crewmembers each year did not wear the devices long enough to be included in the analysis. In Year 1, two crewmembers had daytime (9 a.m.–9 p.m.) wear time of less than 34% and 37% and did not meet the pre-defined wear time threshold of 50% (average wear time across the Year 1 and Year 2 compliant crewmembers was 86%, range 59%–99%) and in Year 2, two crewmembers stopped wearing the device 4 months into the mission. The effects of their exclusion on crew cohesion metrics are unknown.

Notwithstanding the objectivity of proximity data, another major limitation is that it only reveals physical closeness between individuals, not whether individuals are interacting or the nature of such interactions (i.e., positive, neutral, or negative). During a winter-over, each crewmember is assigned a job such as scientist, technician, medical doctor and chef. Certain crewmember responsibilities may make proximity to specific crewmember’s mandatory, affecting the weight of those proximity measures on their cohesion score. While this is a limitation and investigating crewmember responsibilities would provide a more accurate representation of crew cohesion, including an analysis of crew cohesion by job raises risks for participant identifiability. In the same regard, while analyzing results based on participant demographics might provide further understanding of crew cohesion, this was not possible here due to the small sample size and the risk of identifying individual crewmembers. Although face-to-face interactions appear to be the strongest predictor of social and task cohesion when measured with sociometric badges (Zhang et al., 2018), using infrared and vocal features from conversations introduces the risk of a loss of privacy for individuals. That at their current level, actigraphy watches cannot be used to infer the nature of interaction is thus both a limitation and advantage since it does ensure a higher level of privacy for participants; potentially crucial since higher perceived privacy seems related to self-endorsed motivation, more positive relationships with Mission Control, less stress, and higher levels of happiness in crewmembers (Goemaere et al., 2019).

### Conclusion

Unobtrusive proximity measurements were found to provide useful information on crewmembers proximity to each other as a proxy of crew cohesion, and how crew cohesion changes over time in mission in two Antarctic winter-over crews. This technology could be useful for future long-duration spaceflight missions allowing flight surgeons and Psych-Ops to continuously monitor crew interactions and prompt early intervention if a crewmember exhibits signs of social withdrawal from the rest of the crew.

## Data Availability

The datasets presented in this study can be found in online repositories. The names of the repository/repositories and accession number(s) can be found in the article/Supplementary material.
