# Supplementary material for: Proximity measurements for objective assessments of crew cohesion in an antarctic space analog environment
Source: Front Psychol. 2026 Jun 8;17:1783846. doi: 10.3389/fpsyg.2026.1783846 (PMC13283885; doi:10.3389/fpsyg.2026.1783846)
Supplement: Supplementary file 1 [file Supplementary_file_1.docx]

**Supplementary Materials**

Proximity Measurements for Objective Assessments of Crew Cohesion in an Antarctic Space Analog Environment

Mathias Basner^1*^, Ka’alana Rennie^1^, Tyler M. Moore^2^, Ruben C. Gur^2^, Michael G. Smith^1^, Jad Nasrini^1^, Emanuel Hermosillo^1^, Adrian J. Ecker^1^, Victoria Schneller^1^, Christopher W. Jones^1^, Floris P. van den Berg^3^, and Bernd Johannes^4^

^1^Unit for Experimental Psychiatry, Division of Sleep and Chronobiology, Department of Psychiatry, University of Pennsylvania Perelman School of Medicine, Philadelphia, PA, USA

^2^Brain Behavior Laboratory, Department of Psychiatry, University of Pennsylvania Perelman School of Medicine, Philadelphia, PA, USA

^3^Institute of Aerospace Medicine, German Aerospace Center (DLR), Cologne, Germany

^4^European Space Agency

* Correspondence: basner@pennmedicine.upenn.edu


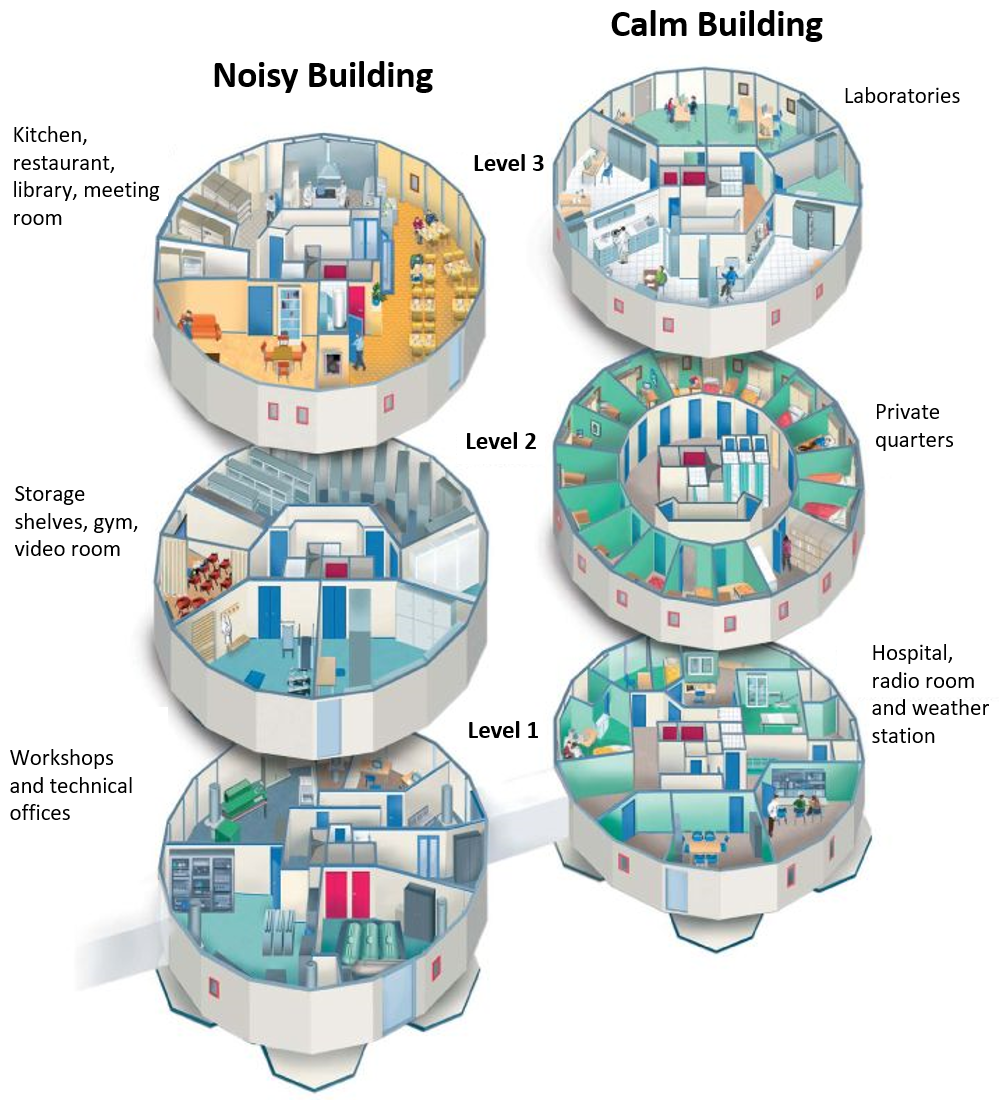


**Supplementary Figure S1: Schematic of Concordia station authorized for use by Institut polaire français Paul-Émile Victor (IPEV). The station consists of two buildings (the container housing the generators is not shown; it can be accessed through the corridor leading away from the noisy building). One is designated as the “calm building”. It houses laboratories, the private quarters, the hospital, a radio room, and the weather station. The other is designated as the “noisy building”. It houses workshops and technical offices, storage shelves, the gym, a video room, the kitchen and restaurant, and a meeting/common room. During the winter-over, each crewmember has his/her own private quarter.**

**Figure S2: Factor analysis of Year 1 crew proximity data by mission half. Numeric values reflect factor analysis loadings associated with each person (values below 0.2 not shown).**

**Figure S3: Factor analysis of Year 2 crew proximity data by mission half. Numeric values reflect factor analysis loadings associated with each person (values below 0.2 not shown).**


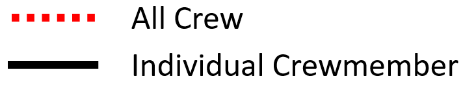

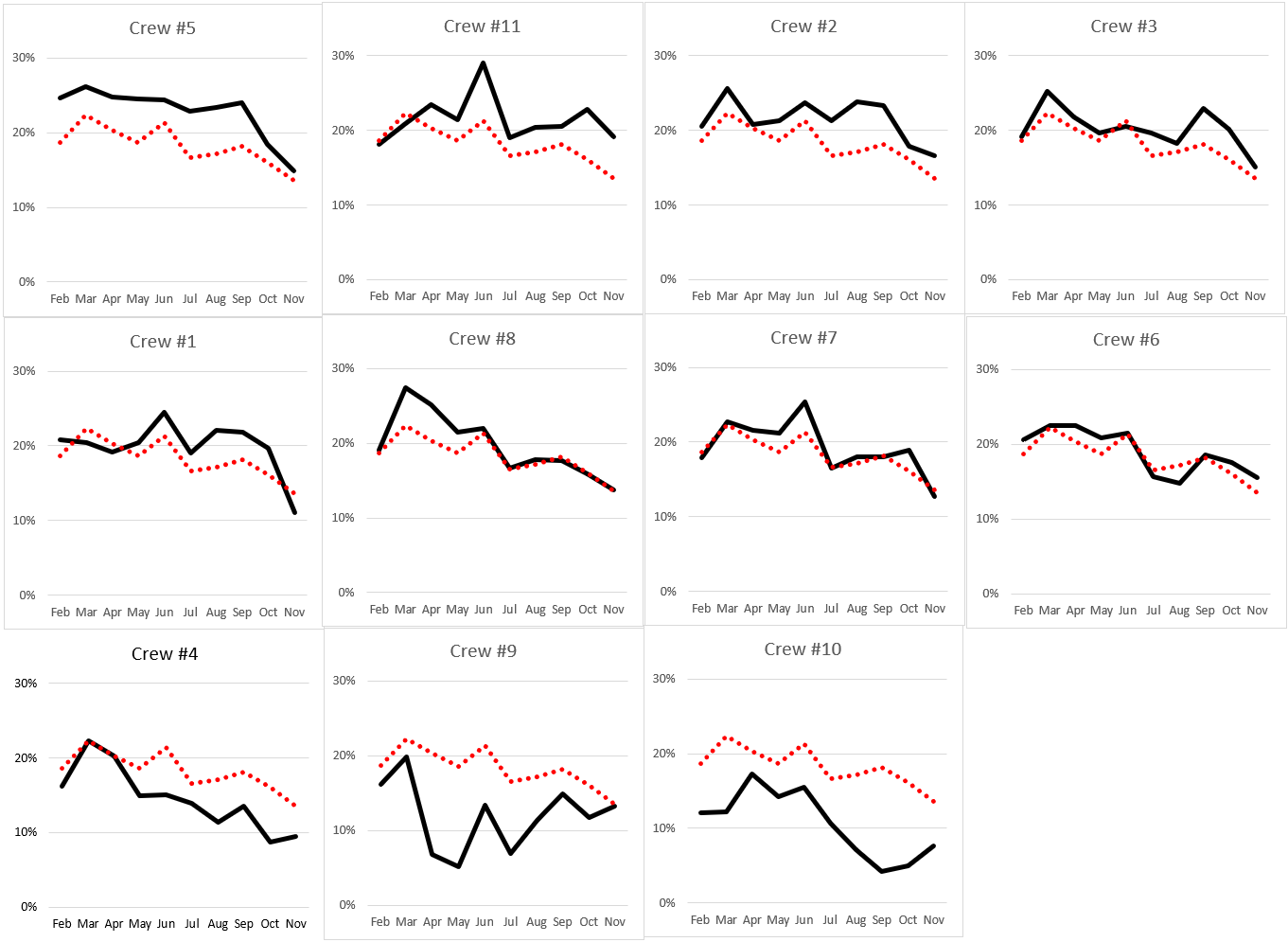


**Figure S4: Comparison of “togetherness” of two individual crewmembers with the average across all crewmembers in Year 1.**


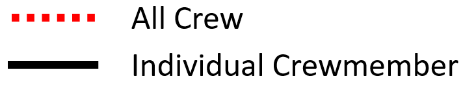

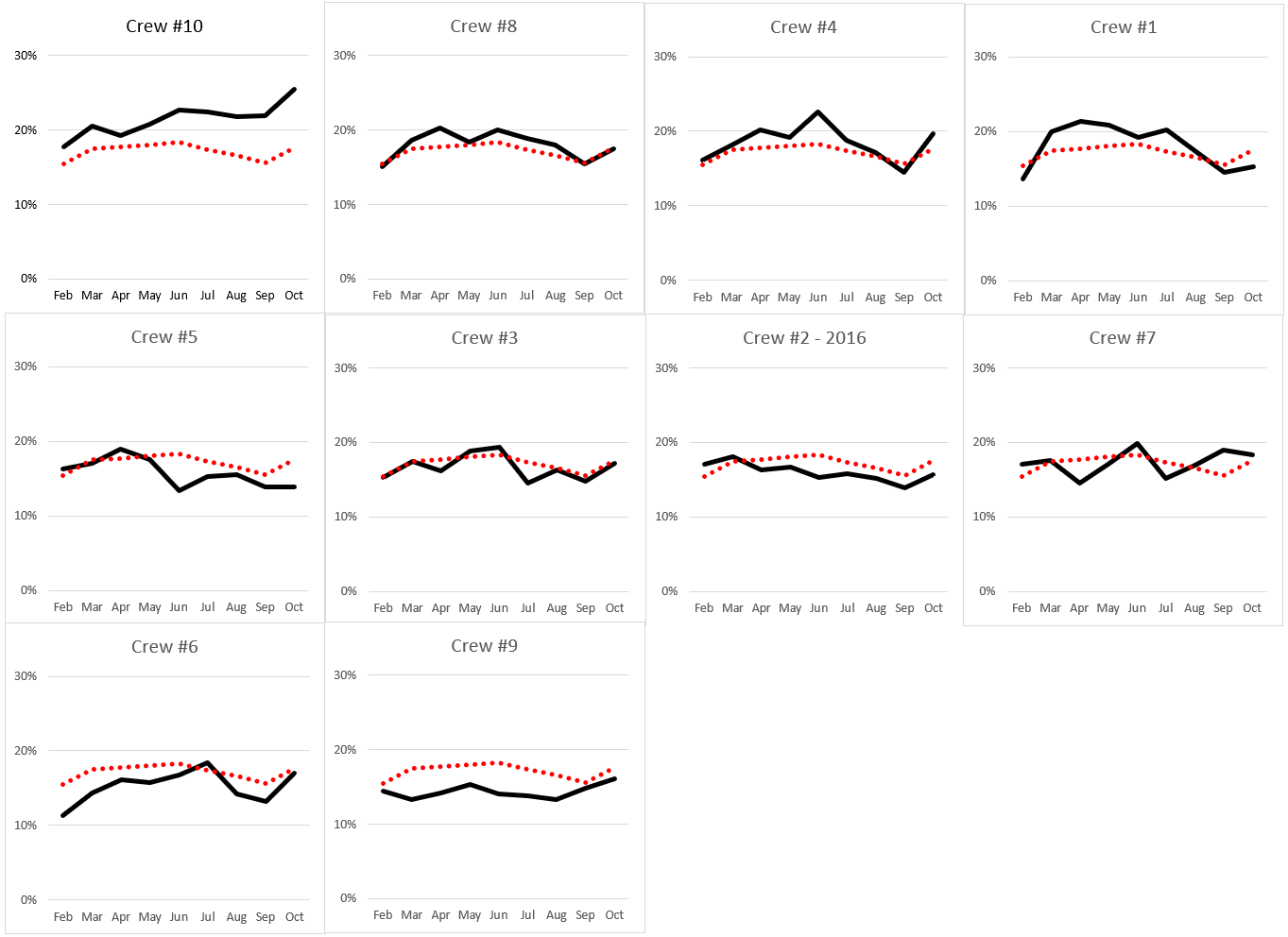


**Figure S5: Comparison of “togetherness” of two individual crewmembers with the average across all crewmembers in Year 2.**
